# Video consultations in medication overuse headache. A randomized controlled trial

**DOI:** 10.1002/brb3.1344

**Published:** 2019-06-17

**Authors:** Svein I. Bekkelund, Kai I. Müller

**Affiliations:** ^1^ Department of Clinical Medicine UiT–The Arctic University of Norway Tromsø Norway; ^2^ Department of Neurology University Hospital of North Norway Tromsø Norway

**Keywords:** headache, health service, management, medication overuse headache, MOH, RCT, telemedicine

## Abstract

**Objective:**

To test the hypothesis that the effect of video consultations is noninferior to traditional consultations in managing patients with overuse headache (MOH).

**Materials and Methods:**

Patients were recruited from referrals to a neurological clinic. In a randomized controlled trial (RCT), headache burden measured by headache impact test (HIT‐6) and frequency of headache days <15 per month and visual analogue pain scale (VAS) at baseline, 3 months and 1 year were compared between groups consulted by video‐ (*n* = 51) and traditional consultations *(n* = 51) in a post hoc analysis.

**Results:**

The overall response rate was 74.5%. HIT‐6 changed from 66.3 (*SD* = 4.7) to 60.0 (*SD* = 9.1) from baseline to 12 months in participants randomized to video consultations and from 65.8 (*SD* = 3.7) to 58.4 (*SD* = 8.3) in the group consulted traditionally (95% CI −2.3 to 6.5, *p* = 0.44). Frequency of headache days <15 per month at 1‐year follow‐up were 9 (23.1%) respectively 10 (27.0%), *p* = 0.60. In the video group, VAS improved by 2.3 points compared to 2.4 in the traditional group from baseline to 12 months (95% CI −1.2 to 1.2, *p* = 0.76). Analyses of repeated measurements comparing HIT‐6 and VAS over two points of time in the two groups were insignificant.

**Conclusion:**

The effect of video consultations is noninferior to traditional consultations in managing MOH patients. Using video may be a good alternative in consulting patients with MOH.

## 1 INTRODUCTION

Medication overuse headache (MOH) is common with a prevalence ranging from 0.5% to 7.2% reported in population studies, and is estimated to cause about half of chronic headache cases (Aaseth et al., 2008; Westergaard, Glumer, Hansen, & Jensen, 2014). MOH, a condition that largely could be avoided, is disabling and costly to both individuals and society (GBD, 2015, Neurological Disorders Collaborator Group, 2017; Linde et al., 2012). Since the presence of migraine or another primary headache disorder is a necessary precursor for the development of MOH, this add‐on headache is an important focus for prevention and reduction of headache‐related disability (Munksgaard & Jensen, 2014). Patient education and preventive treatment are important steps in the management of these patients (Diener, Holle, Solbach, & Gaul, 2016). MOH is a common group of headache patients among those referred to specialist for second opinion from general practitioners (Muller, Alstadhaug, & Bekkelund, 2016a). It is, however, important to be aware that consequences of painkiller use in headache patients may be unrecognized (Bekkelund & Salvesen, 2002). The costs per visit for rural headache patients visiting a headache specialist in North Norway were estimated to €249 (travel cost) and €234 (loss of income; Muller, Alstadhaug, & Bekkelund, 2016b). Furthermore, acceptability and feasibility using video in consulting nonacute headache patient were favourable (Muller et al., 2016b).

Access to care and patient's preferences are important elements to consider when organizing an optimal service for the patients. Access variability for headache sufferers referred to a specialist is a recognized problem (Tassorelli, Farm, et al., 2014). A cross‐sectional study showed that MOH patients (*n* = 65) preferred information to be given personally (Munksgaard et al., 2011). They also preferred consultations by telephone to e‐mail or other sources of information, but video consultations were not evaluated (Munksgaard et al., 2011). Lack of well‐documented treatment standards and best practice strategies add to the complexity in the management of the disorder (de Goffau, Klaver, Willemsen, Bindels, & Verhagen, 2017). In this study, we investigated whether use of video consultations is effective in the treatment of patients with MOH referred for second opinion to a neurological outpatient department. The primary hypothesis was that the treatment effect of video consultations is noninferior to traditional consultations in managing MOH patients.

## 2 MATERIALS AND METHODS

### 2.1 Study design and patients

**Figure 1:**
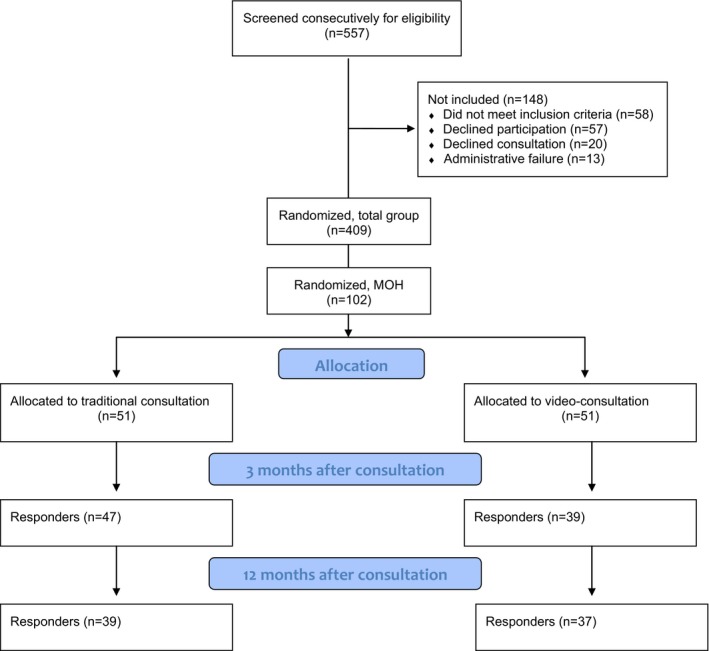
Flowdiagram of participants throughout the study

The present study is secondary research based on a noninferiority randomized controlled trial (RCT) comparing treatment outcomes between groups of headache patients referred to Neurologist consulted by using video and in the traditional manner. All consultations were organized and completed at the Department of Neurology in Tromsø University Hospital, Norway, which is the only neurological specialist center in the region (Northern part of Norway including Svalbard). Altogether, the hospital served 268,511 inhabitants (December 2014) living in an area of 140,541 km^2^ according to Norway Statistics. In the study period (30 September 2012 until 30 March 2015), we consecutively screened 557 and enrolled 402 in the study. Of them, *n* = 102 had a diagnosis of MOH (Figure 1).

A study nurse received the patients at the entrance, controlled the self‐administered prefilled questionnaires, Headache Impact Test‐6 (HIT‐6, Yang, Rendas‐Baum, Varon, & Kosinski, 2011), a horizontal visual analogue scale (VAS) ranging from 0 to 10; 0 = no pain, 10 = worst possible pain (Lundqvist, Benth, Grande, Aaseth, & Russell, 2009), and consent forms and then called the external Research Department at the University Hospital for block randomization. A computerized randomization using an Rnd function in Microsoft Access was performed before patients were guided either to a patient examination room at the department (traditional group) or to a videoconference room located next to it (video group) out of examiners view. The videoconference room had a Cisco C40 integrator package installed providing a direct and encrypted two‐way video and audio communication between neurologist and patient (Muller et al., 2016b). The neurologists conducted remote visits via a Cisco EX60 unit from the examination room where also the traditional face‐to‐face consultations took place. Two neurologists (KIM and SIB) carried out all consultations without neurological examinations, which was initiated by check of inclusion criteria and completion of diagnostic classification.

Norwegian‐speaking males and females aged 16–65 years referred to the Department of Neurology from primary care for management of MOH with maximum 4 months waiting time to reduce the risk of exclusions by time limitation and to adapt to the Department's waiting list, were accepted for inclusion. All patients had chronic headache (headache ≥15 days per month for more than 3 months) and at least one primary headache disorder classified by one of the study investigators (KIM and SIB; ICHD, 2004). All fulfilled the revised criteria of MOH classification (use of simple analgesics ≥15 days per month or acute medication, triptans, ergots, opioids or combination analgesics ≥10 days per month for over 3 months; Olesen et al., 2006). Those with abnormal findings on either clinical neurological examination reported by the referring doctor or by brain imaging suggestive of secondary etiology and being examined by a specialist for headache within 2 years prior to inclusion time were excluded to ensure that only naïve patients with new onset headache were included.

### 2.2 Outcome variables

Data were collected at baseline (consultations by KIM and SIB) and at 3 and 12 months (questionnaires) with a reminder after 2 weeks to the nonresponders. We sent a structured questionnaire by patient preference, either through an online survey service (Questback) or by letter mail. ∆HIT‐6 was selected as primary endpoint and frequency of headache days <15 per month, ∆VAS, frequency of reduction to episodic headache, HIT‐6 reduction ≥5 and HIT‐6 increase ≥5 as secondary endpoints. Clinical and headache characteristics including diagnostic classification according to International Classification of Headache Disorders‐2 ++(ICHD, 2004) of the participants were recorded. The patients responded to “number of headache days per month for the last three months” to classify chronic headache. Use of painkillers (OTC and prescriptive) and use of triptans were ticked off on the registration form. Treatment options and advices recommended by the specialist were evidence based (Evers & Jensen, 2011), and supplied by general advices as followed: Withdrawal of medication, reduction in use of medication (misuse of eg, triptans, patient denying complete withdrawal), preventive treatment, triptan use, physical training, trigger avoidance, regular sleep, weight reduction, menstruation advises (use of long‐lasting triptans before and during menstruation periods, consulting primary physician to regulate or postpone menstruation cycles if suitable).

All patients were brain scanned, either by CT (spiral technique with reconstructions in coronal, sagittal, and axial planes) or by 1.5T MRI machines with a Head/SENSE‐Head/Flex‐L coil. All MRI images were presented with sagittal 3D fluid‐attenuated inversion recovery, axial T2 turbo spin echo 4 mm, axial T2 fast field echo 4 mm, axial diffusion 4 mm, and sagittal T1 spin echo 5.5 mm. Experienced radiologists or neuroradiologists evaluated the images. Neuroimaging findings were classified as “normal,” “nonsignificant,” or “significant” (Sempere et al., 2005).

### 2.3 Statistical analyses

We used SPSS 23 for data analyses. Gaussian distribution of continuous variables was confirmed by inspection of histograms and by calculating kurtosis and skewness. Descriptive variables are presented as mean (*SD*) or numbers (%). Categorical variables were compared by chi‐square test and continuous data by two‐sided Student *t* test with *p* < 0.05 as level of significance. Comparisons between variables with small numbers are labeled “not applicable” (NA). We used mixed between‐within participants ANOVA to assess whether change in HIT‐6 and VAS differed between telemedicine and traditional consultations prospectively over three time points. These results are shown in figures and presented with F scores and significance levels. Details about power estimation are published elsewhere (Muller et al., 2016a).

### 2.4 Consent, registration and ethics

Oral and written consent were obtained from all participants before data collection. The Norwegian National Committee for Medical and Health Research Ethics (REC), number 2009/1430/REK approved the study. The trial was registered at the Norwegian Research and Management database (ID3897/HST959‐10) and at ClinicalTrials.gov (id. NCT02270177).

## 3 RESULTS

From a total population of 557 headache patients referred to specialist for second opinion, 102 classified as MOH (18.3%) fulfilled the inclusion criteria and were included and randomized to either video consultations (*n* = 51) or traditional consultations (*n* = 51; Figure 1). At 3‐month follow‐up, 47/51 (92.2%) in the video group and 39/51 (76.5%) in the traditional group responded (*p* = 0.054), giving a total response rate of 86/102 (84.3%). The equivalent responses at 1‐year follow‐up were 39/51 (76.5%) in the video group, 37/51 (72.5%) in the traditional group (*p* = 0.67), and 76/102 (74.5%) overall. Table 1 shows social and demographic data in comparison between patient groups consulted by video and traditionally. No statistically significant differences between the groups were found. Also, waiting time to specialist and consultation time were similar, and there was no significant change in frequency of sick leave from baseline to 1 year (Table 1).

**Table 1:** Headache characteristics at baseline and at 1‐year follow‐up in randomized groups of patients with medication overuse headache referred to specialist for second opinion

	Baseline			1‐year follow‐up		
	Video (*n* = 51)	Traditional (*n* = 51)	*p* value	Video (*n* = 39)	Traditional (*n* = 37)	*p* value
Age (years)	40.5 (11.7)	39.1 (13.8)	0.56	40.6 (11.5)	37.7 (14.4)	0.33
Females	38 (74.5)	39 (76.5)	1.0	29 (74.4)	30 (81.1)	0.59
Age at headache onset (years)	23.3 (14.7)	23.0 (12.9)	0.93	22.2 (15.3)	23.2 (13.9)	0.78
Headache duration (year)	18.0 (13.4)	16.5 (13.3)	0.56	19.6 (14.0)	15.4 (13.4)	0.18
Consultation time (minutes)	44.3 (8.0)	46.9 (11.3)	0.19			
Primary headaches						
Migraine	50 (98.0)	48 (94.1)	0.62	38 (97.4)	34 (92.0)	0.35
Tension type headache	40 (78.4)	41 (80.4)	1.0	31 (78.5)	30 (81.1)	1.0
Normal MRI	39	33	1.0			
Normal CT	12	18	0.46			
Painkillers (over‐the‐counter)	46 (90.2)	48 (94.1)	0.72	33 (84.6)	34 (91.9)	0.48
Painkillers (prescription)	16 (31.4)	14 (27.5)	0.83	11 (28.2)	8 (21.6)	0.60
Painkillers daily	20 (39.2)	18 (35.3)	0.28	14 (35.9)	9 (24.3)	0.15
Triptans	22 (43.1)	26 (51.0)	0.87	20 (51.3)	21 (56.7)	0.75
Triptans daily	4 (7.8)	1 (2.0)	NA	1 (2.6)	0	NA

Note

Data are presented as mean (*SD*) or numbers (%).

Abbreviation: NA, not applicable.

Most common primary headaches classified in the participants and uses of medications are presented in Table 2. All patients had normal MRI or CT of the brain before the consultation (Table 2). Use of painkillers was only minimally reduced at 1‐year follow‐up compared to baseline, while triptan use had increased slightly (Table 2). Treatment and specific advices are listed in Table 2. In the video group, withdrawal or reduction of medication use were recommended in 47 (92.1%) compared to 45 (88.3%) in the others, *p* = 0.58.

**Table 2:** Treatment and advices to patients with medication overuse headache initiated by neurologist at baseline

	Video (*n* = 51)	Traditional (*n* = 51)	*p* value
Reduction of acute medication	30 (58.8)	26 (51.0)	0.55
Withdrawal of acute medication	17 (33.3)	19 (37.3)	0.84
Preventive treatment*	40 (78.4)	33 (64.7)	0.19
Antihypertensives	12 (23.5)	13 (25.5)	1.0
Antiepileptics	10 (19.6)	10 (19.6)	1.0
Antidepressants	20 (39.2)	12 (23.5)	0.14
Triptans	13 (25.5)	12 (23.5)	1.0
Physical training	19 (37.3)	21 (41.2)	1.0
Trigger avoidance	11 (21.6)	20 (39.2)	0.08
Regular sleep	2 (3.9)	9 (17.6)	NA
Weight reduction	1 (2.0)	0	NA
Menstruation advices	1 (2.0)	0	NA

Note

Some patients received more than one drug, numbers (%).

Abbreviation: NA, not applicable.

* Indicates any preventive drug.

Table 3 shows endpoint variables in the two groups at baseline, 3 months and 12 months. Mean HIT‐6 was reduced by 6.3 (9.5%) in the video group and 7.4 (11.2%) in the traditional group from baseline to 12 months (95% CI −2.3 to 6.5, *p* = 0.44). In any group, frequency of patients with <15 headache days per month in the follow‐up period varied between 21.3% and 27.0% (Table 3). VAS improved 2.3 points (31.5%) at average (video group) and 2.4 (traditional group; 33.3%) in the same period (95% CI −1.2 to 1.2, *p* = 0.76). All comparisons are insignificant (Table 3). Likewise, about 40% in both groups reported <15 headache days per month during the last 3 months at 3‐month follow‐up while the frequency of patients with episodic headache at 1‐year follow‐up was reduced to 26.6% (video group) and 32.4% (traditional group; Table 3). Pooled data showed an episodic headache pattern in 41.9% at 3 months and 28.9% at 1 year.

**Table 3:** Endpoint variables at 3 months and at 1‐year follow‐up in randomized groups of patients with medication overuse headache referred to specialist for second opinion

	Baseline			3‐month follow‐up			1‐year follow‐up		
	Video (*n* = 51)	Traditional (*n* = 51)	*p* value	Video (*n* = 47)	Traditional (*n* = 39)	*p* value^a^	Video (*n* = 39)	Traditional (*n* = 37)	*p* value^a^
HIT‐6	66.3 (4.7)	65.8 (3.7)	0.56	58.7 (9.0)	61.5 (6.8)	0.12	60.0 (9.1)	58.4 (8.3)	0.44
∆HIT‐6				7.4 (10.3)	4.8 (8.0)	0.21	6.6 (11.2)	7.8 (8.7)	0.63
<15 headache	0	0		10 (21.3)	10 (25.6)	0.80	9 (23.1)	10 (27.0)	0.60
Days per month									
<7 headache	0	4		0	3		0	2	
Days per month									
HIT‐6 reduction ≥5				29 (61.7)	15 (38.5)	0.051	11 (21.6)	11 (29.7)	0.80
HIT‐6 increase ≥5				7 (15.0)	3 (7.7)	0.34	6 (11.8)	2 (3.9)	0.27
VAS	7.3 (2.0)	7.2 (2.4)	0.86	4.3 (2.9)	4.9 (2.2)	0.29	5.0 (2.6)	4.8 (2.5)	0.76
∆VAS				3.0 (3.3)	2.3 (3.2)	0.32	2.4 (3.2)	2.5 (3.8)	0.95
Episodic headache	0	0		19 (40.4)	17 (43.6)	0.80	10 (26.6)	12 (32.4)	0.54

Note

Data are presented as mean (*SD*) or numbers (%)

Abbreviations: HIT‐6, Headache impact test; VAS, Visual analogue scale.

a Compared to baseline.

**Figure 2:**
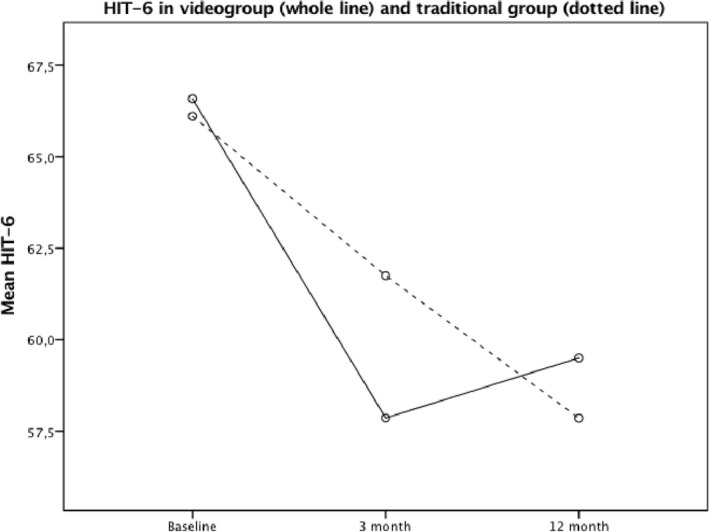
Comparisons of HIT‐6 over three time points between headache patients randomized to video consultations and traditional consultations. There were no differences between the groups (mixed design ANOVA; *F*(2, 62) = 0.381, *p* = 0.54)

**Figure 3:**
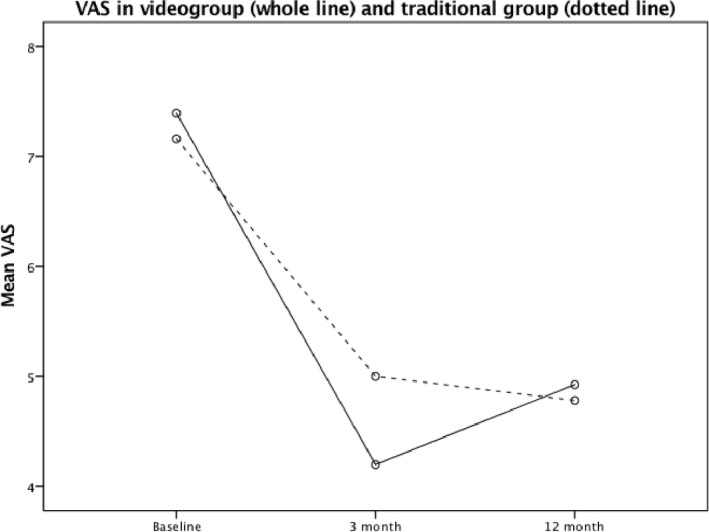
Comparisons of visual analogue scale (VAS) over three time points between headache patients randomized to video consultations and traditional consultations. There were no differences between the groups (mixed design ANOVA; *F*(1, 56) = 0.117, *p* = 0.73)

Estimation of reliability, HIT‐6 Cronbach alpha coefficients were 0.82, 0.79, and 0.78 at baseline, 3 month and 1 year, respectively. Mixed between‐within participants ANOVA analysis showed no significant differences in HIT‐6 or VAS between the video group and the traditional group measured prospectively at three time points, *F*(2, 62) = 0.381, *p* = 0.54 and *F*(1, 56) = 0.117, *p* = 0.73 for HIT‐6 and VAS, respectively (Figures 2 and 3).

## 4 DISCUSSION

The results from this RCT trial document that video consultations are noninferior to traditional face‐to‐face consultations in treating MOH. The condition improved until 3 months, then deteriorated slightly at 1‐year examination in both groups. At 1‐year assessment, HIT‐6 improved by approximately 10% and VAS about 30% compared to baseline in both groups. After 3 months, 40%/43% (video/traditional groups), respectively 27%/32% at 1‐year observation had an episodic pattern of headache. Use of video consultations is therefore a good alternative in consulting patients with MOH.

Treatment strategies for MOH are largely based on experience and consensus (Evers & Jensen, 2011), and scientific evidence exists only to a limited extent (Chiang, Schwedt, Wang, & Dodick, 2016). Neither is it reported similar studies like the present one investigating the efficacy of using electronic devices in consulting MOH patients despite the existence of guidelines for teleheadache consultations (Wechsler et al., 2013). Beyond stroke, there are only a few randomized controlled telemedicine trials in neurological conditions in general (Chua, Craig, Wootton, & Patterson, 2001; Rubin, Wellik, Channer, & Demaerschalk, 2013), and headache in particular (Muller et al., 2016a). Thus, telemedicine is widely used in neurological clinics as well as for headache management (Davis, Coleman, Harnar, & King, 2014; George et al., 2012; Yurkiewicz et al., 2012).

In a previous study where new neurological patients (including headache) were randomized to either telemedicine or face‐to‐face consultations in an outpatient specialist center, feasibility, patient' satisfaction, and number of drug prescriptions were similar in both groups (Chua et al., 2001). In contrast, patients in the telemedicine group had more investigations and were more concerned about confidentiality and embarrassment (Chua et al., 2001). A Cochrane report reviewing 93 eligible telemedicine trials until June 2013 with a wide range of conditions (other than neurological) concluded that cost and acceptability by patients and healthcare are uncertain due to limited data available (Flodgren, Rachas, Farmer, Inzitari, & Shepperd, 2015). Disease severity and purpose of the intervention (eg, monitoring chronic diseases, diagnose new patients) are some factors that may influence the outcome (Flodgren et al., 2015). Recently, it is shown that video consultations for new patients with primary, nonacute headaches referred from general practice to specialist are cost saving, feasible, satisfying for the patients, effective and safe in a mixed rural and urban population (Muller et al., 2016b; Muller, Alstadhaug, & Bekkelund, 2017a, 2017b). In line with this, the present secondary analyses of the MOH subgroup shows that after 12 months monitoring of treatment effect, neither headache burden nor pain level were statistically in favor of traditional consultations (Figures 2 and 3). Consequently, patients with difficult headache may benefit from use of information and communication technologies in consultation with headache specialists, and should therefore undergo further investigations.

Methodologically controversies associated with MOH make comparisons with others difficult. Changes in definition over time and invalid classification of the preexisting primary headache disorder(s) may contribute to data uncertainty (Ferrari, Coccia, & Sternieri, 2008). In a previous Norwegian study, headache days was reduced by 32% and medication days by 46% in a group randomized to brief intervention (BI; advices and education) compared to the control group treated by “standard of care” in general practice (Kristoffersen et al., 2015). Headache specialists gave BI as a 1‐day course. This study demonstrates the importance of a systematic approach in the treatment of MOH. In the present study, we administered advices by a structured interview that included discontinuation of overused medicine and supplementary preventive medication to the migraineurs as recommended (Chiang et al., 2016). The results are within range of a clinically important difference for HIT‐6 and VAS (Coeytaux, Kaufman, Chao, Mann, & Devellis, 2006; Hawker, Mian, Kendzerska, & French, 2011). In a multicenter study, 46% reversed to episodic headache 6 months after intervention (Tassorelli, Jensen, et al., 2014). Previous studies report a 1‐year relapse rate of 40% (Katsarava et al., 2005). Despite differences in definition, primary endpoints, and the fact that trials comparing treatment options in MOH are largely performed in tertiary centers, our results are comparable (Katsarava, Limmroth, Finke, Diener, & Fritsche, 2003).

Continuously enrollment of patients referred to specialist for second opinion within a defined geographical area where all specialists are centralized into one hospital, strengthen the generalizability of the sample and minimizes dropouts by providing full control over patient's logistics. Another favorable aspect is the optimal balance between the groups in the trial as far as size and background variables concerns, which was created by the randomization procedure. Contrary, the secondary research setting increases the risk of statistical type 2 failures due to underpowered sample size. Furthermore, the common location at the hospital where all the consultations took place may have biased the results. Another uncertainty is lack of information about reason(s) why withdrawal or reduction of medication was not recommended in 9%. This is discussed more detailed elsewhere (Muller, Alstadhaug, & Bekkelund, 2017b).

## 5 CONCLUSIONS

The pattern of treatment responses and long‐term treatment outcome after one‐time consultation for patients with MOH were similar in both groups providing evidence that video consultations are noninferior compared to traditional consultations in treating these patients. Consulting MOH patients by using video devices is an option to consider in suitable areas. The high frequency of MOH among patients referred from general practice to specialist, demonstrates a need for easy access to second opinion for this patient group.

## CONFLICT OF INTEREST

The authors declare no conflict of interest.

## Data Availability

The data that support the findings of this study are available from the corresponding author upon reasonable request.
